# Transcription Factor XBP1s Impairs Endometrial Receptivity by Promoting ENO1-Mediated Glycolysis and Lactate Production

**DOI:** 10.3390/cells15181656

**Published:** 2026-09-14

**Authors:** Kangkang Gao, Mengqi Si, Huijun Wang, Huijie Zhang, Feng Zhu, Pengfei Lin, Huatao Chen, Xinhua Zheng, Yaping Jin

**Affiliations:** 1 School of Medicine, Pingdingshan University, Pingdingshan 467000, China; 2 Key Laboratory of Animal Biotechnology of the Ministry of Agriculture and Rural Affairs, College of Veterinary Medicine, Northwest A&F University, Xianyang 712100, China

**Keywords:** endometrial receptivity, XBP1s, glycolysis, lactate, ENO1, embryo implantation

## Abstract

Successful embryo implantation necessitates formation of a complex and tight connection between a well-developed embryo and highly receptive endometrium. Type I interferon IFN-τ is a pregnancy recognition signal in ruminants that promotes the establishment of endometrial receptivity. However, the mechanisms that underpin this process largely remain unknown. Previous CUT&Tag assays revealed that XBP1s preferentially targets genes in the glycolytic pathway. In this study, we found that IFN-τ significantly inhibited glycolysis and lactate production during peri-implantation. In addition, overexpression of *XBP1s* reversed the inhibition of glycolysis by IFN-τ and promoted expression of glycolysis rate-limiting enzymes (HK1, PFK-1, and PKM1) and lactate production. Dual-luciferase reporter and electrophoretic mobility shift assays demonstrated that XBP1s directly binds to the promoter of the ninth step gene of glycolysis, *ENO1*. Moreover, ENO1 and XBP1s exhibited congruent expression patterns, and both factors also reversed IFN-τ-induced endometrial receptivity and inhibition of glycolysis. In contrast, knockdown of *ENO1* enhanced the effects of IFN-τ. Interestingly, lactate also inhibited the establishment of endometrial receptivity and PGES expression. In conclusion, our data suggest that XBP1s negatively regulates endometrial function through the transcriptional regulation of ENO1-promoted lactate production. This study provides key insights into the mechanisms by which XBP1s acts in female reproductive development.

## 1. Introduction

Early pregnancy failure is one of the most important causes of low reproduction rates in livestock and is a major hindrance to the development of livestock farming [1]. Successful embryo implantation requires a complex and tight connection between a well-developed embryo and a highly receptive endometrium [2]. During embryo implantation in ruminants, the endometrium undergoes morphological and functional changes, mainly through the action of estradiol (E_2_), progesterone (P_4_), and type I interferon IFN-τ to provide the best environment for implantation of the conceptus [3]. IFN-τ is secreted by mononuclear trophectoderm cells, which act as the maternal recognition signal for ruminants [4]. IFN-τ is secreted from day 10 of gestation (day 0 = day of estrus) and persists until day 21–25, with a maximum peak on day 14–16 in sheep. IFN-τ inhibits the pulsatile release of endometrial prostaglandin F_2α_ (PGF_2α_) by suppressing the transcription of estrogen receptor alpha (ESR1) and oxytocin receptor (OXTR), thereby regulating endometrial receptivity [5]. IFN-τ also promotes endometrial expression of prostaglandin E_2_ receptor 2 (EP2) and 4 (EP4) [6]. Prostaglandin precursors in the estrous cycle tend to be synthesized toward PGF_2α_, whereas synthesis of these precursors gravitates toward prostaglandin E_2_ (PGE_2_) during pregnancy. PGE_2_ is secreted paracrinely in the corpus luteum to protect it from lysis [7]. In addition, IFN-τ binds to the interferon α/β receptor (IFNAR) to activate the expression of classical or non-classical interferon-stimulated genes (ISGs) [8].

The endoplasmic reticulum (ER) is the key organelle responsible for protein synthesis and folding. ER stress (ERS) is triggered when accumulation of unfolded or misfolded proteins occurs in the organelle [9]. We reported previously that GRP78, a major regulator of ERS, was activated at the site of implantation on day 5 of embryo attachment in pregnant mice [10]. Moreover, inhibition of ERS improves the endometrial development and receptivity required for implantation during early pregnancy in dairy cows [11]. However, the mechanism by which ERS impacts endometrial cell function during the peri-implantation period of pregnancy in ruminants is unknown.

X-box binding protein 1 splicing variant (XBP1s) is the key transcription factor involved in ERS and in monitoring of protein folding. When ERS occurs, ER-sensing factors, including inositol-requiring enzyme 1 (IRE1), are activated. Activated IRE1 possesses endonuclease activity and splices *XBP1* mRNA to generate active forms of XBP1s [12]. XBP1s enhances the proliferative and antioxidant abilities of goat spermatogonial stem cells through a mechanism that involves regulation of these cells by macrophages [13]. Furthermore, the expression of *XBP1s* was detected at all stages of porcine somatic cell nuclear transfer embryo development [14]. In addition, knockdown of *XBP1s* decreased viability and triggered cell death in porcine embryonic fibroblast cells, decreased the in vitro developmental competence of bovine embryos, and a loss-of-function mutation in XBP1s caused embryonic lethality in mice [15]. Studies have also shown that XBP1 exhibits a spatiotemporal expression pattern in the mouse endometrium during early pregnancy, participating in embryo implantation and decidualization [16]. Moreover, elevated serum XBP1 levels are associated with an increased risk of early pregnancy loss in humans, and under ERS conditions, XBP1s has been shown to regulate mitochondrial dysfunction and autophagy in decidual stromal cells through the TRAF6/mTORC2 axis, further underscoring its critical role in the maintenance of early pregnancy [17,18]. Therefore, we hypothesize that XBP1s is essential for reproduction in females, although the effects of XBP1s on endometrial function during the peri-implantation period of ruminant pregnancy and the mechanisms that are involved remain unclear.

Glycolysis is a fundamental pathway in cellular energy metabolism that mediates the conversion of glucose to pyruvate and then to lactate by a cascade of enzymes. The main rate-limiting enzymes in the pathway are hexokinase 1 (HK1), phosphofructokinase 1 (PFK-1), and pyruvate kinase muscle isoform 1 (PKM1) [19]. Lactate acts as a signaling molecule in the regulation of cellular metabolism by promoting glycolysis and enhancing cellular adaptation to the environment. In certain cancers, lactate increases the transcription and activation of proteolytic enzymes, including matrix metalloproteases and cathepsins involved in the breakdown of the extracellular matrix [20]. Moreover, lactate induces angiogenesis, endothelial cell migration, and tube/vessel formation via the recruitment of vascular endothelial growth factor (VEGF) and activation of NF-κB/IL-8 signaling [21,22]. Furthermore, lactate modulates the function of immune cells, including macrophages, cytotoxic T cells, and monocytes, thereby reducing production of proinflammatory cytokines while simultaneously increasing release of immunosuppressive factors [23,24,25]. An expanding number of studies have shown that lactate, as an end product of glycolysis, is also involved in diverse physiological and pathological processes. However, the effect of lactate on endometrial function in ruminants remains unclear. In the present study, we evaluated changes in the expression of XBP1s during different periods of goat pregnancy, as well as the alterations in endometrial glycolysis and lactate levels during the peri-implantation period. This analysis clarified the effects and mechanisms of action of both transcription factor XBP1s and lactate on endometrial function. The data also provide a rigorous basis for solving the problems of low reproduction and high culling rates in livestock farming.

## 2. Materials and Methods

### 2.1. Cell Culture and Drug Treatment

Immortalized goat endometrial epithelial cells (gEECs) were identified and preserved previously in our laboratory [26]. HEK 293T cells were obtained from the cell bank of the Typical Culture Preservation Center of the Chinese Academy of Sciences (Shanghai, China). Furthermore, gEECs and HEK 293T cells were cultured separately in 10% fetal bovine serum (FBS; Gibco Life Sciences, Grand Island, NY, USA) and 1% antibiotic–antimycotic (containing penicillin, streptomycin, and amphotericin B; Invitrogen, Inc., Carlsbad, CA, USA) in DMEM/F12 and high-glucose DMEM at 37 °C in a humidified environment containing 5% CO_2_. The medium was replaced with fresh medium when the cell confluence reached 70–80% and the following treatments were performed: (1) IFN-τ (40 ng/mL; C600063-0010; Sangon Biotech Co., Ltd., Shanghai, China) for 6 h; or, (2) L-lactate (20 mM; T4845; TargetMol, Boston, MA, USA) was pretreated for 2 h before IFN-τ was added for 6 h [27].

### 2.2. Plasmid Construction

The pcDNA3.1-XBP1s and gEECs-shXBP1s cell lines were previously preserved in our laboratory [26,28]. The *ENO1* gene fragment was amplified using primers (Table 1) and gEECs cDNA as a template and was cloned into the pcDNA3.1 vector using a seamless cloning method (ClonExpress II One Step Cloning Kit, Vazyme, Nanjing, China) according to the manufacturer’s instructions to produce pcDNA3.1-ENO1. The pcDNA3.1-XBP1s or pcDNA3.1-ENO1 plasmids were transfected using TurboFect (Thermo Fisher Science, Waltham, MA, USA) according to the manufacturer’s protocol when the cell confluence reached 70% in six-well cell culture plates. The empty vector was used as a negative control. Transfected cells were incubated for 12 h before the next step.

### 2.3. ENO1 siRNA Transfection

The siRNA targeting the goat *ENO1* mRNA sequence (si-ENO1) and the non-targeting siRNA (si-NC) were synthesized by GenePharma (Shanghai, China) (Table 2). Cell transfection was performed as described in Section 2.2.

### 2.4. Protein Extraction and Western Blotting

The treated gEECs were collected, and whole-cell proteins were extracted using the KGP2100 kit (KeyGEN Biotech, Jiangsu, China) according to the manufacturer’s protocol. Protein concentrations were determined using the bicinchoninic acid assay (KGPBCA; KeyGEN Biotech) followed by SDS-PAGE and immunoblotting, as described previously [26]. The membrane was probed with anti-XBP1 antibody (diluted 1:1000; ab220783, Abcam, Cambridge, UK), anti-PTGS2 antibody (1:1000; CY8852, Abways, Shanghai, China), anti-PGES antibody (1:1000; ab62050, Abcam), anti-HK1 antibody (1:1000; 2024, Cell Signaling Technology, Boston, MA, USA), anti-PFK-1 antibody (1:1000; 55028-1-AP, Proteintech Group, Inc., Wuhan, China), anti-PKM1 antibody (1:1000; 15821-1-AP, Proteintech), anti-GLUT1 antibody (1:1000; 21829-1-AP, Proteintech), anti-G6PC3 antibody (1:1000; ab221647, Abcam), or anti-β-actin antibody (1:2000; HRP-66009, Proteintech) overnight at 4 °C. The secondary antibody (Zhongshan Golden Bridge Biotechnology, 1:5000) coupled with horseradish peroxidase (HRP) was incubated at room temperature for 1 h. Finally, the protein bands were visualized using a gel imaging system (Tanon Biotech, Shanghai, China) and were examined with Quantity One software version 4.6.8. (Bio-Rad Laboratory, Hercules, CA, USA) for quantification.

### 2.5. RNA Extraction and RT-qPCR

The treated gEECs were collected, and total cellular RNA was extracted using TRIzol (TaKaRa Bio, Inc., Dalian, China). The cDNA was synthesized by reverse transcription using the PrimeScript™ RT reagent Kit with gDNA Eraser (TaKaRa) following the manufacturer’s instructions. RT-qPCR was performed using ChamQ SYBR qPCR Master Mix (Vazyme Biotech Co., Ltd., Nanjing, China) according to the manufacturer’s procedure using Bio-Rad CFX96 (Bio-Rad Laboratories, Inc., Hercules, CA, USA) and the appropriate primers (Table 1). The gene for *β-actin* was used as an invariant control, and the relative expression of each gene was determined using the 2^−∆∆Ct^ method.

### 2.6. Dual-Luciferase Reporter Assay System

Dual-luciferase reporter assays were performed as described previously [29]. Briefly, the promoter sequence of the *ENO1* gene (−2001 to −1) was amplified from gEEC genomic DNA using the primer sequences shown in Table 1. The 2001 bp fragment was cloned into the PGL4.10 vector using a seamless cloning method (ClonExpress II One Step Cloning Kit) to produce PGL4.10-ENO1. The PGL4.10 or PGL4.10-ENO1 and pRL-CMV luciferase reporter plasmids were co-transfected with pcDNA3.1-XBP1s into HEK 293T cells using TurboFect, and the cells were collected after 36 h. Transfectants were analyzed using the Dual-Luciferase Reporter Assay System (Promega, Madison, WI, USA) and SPARK Multimode Microplate Reader (Tecan, Mannedorf, Switzerland) to determine luciferase activity according to procedures provided by the manufacturers. Renilla luciferase was used as the normalization control.

### 2.7. Electrophoretic Mobility Shift Assays

Electrophoretic mobility shift assays (EMSA) were performed as outlined previously [29]. Briefly, gEECs were transfected with pcDNA3.1-XBP1s, and nuclear proteins were extracted using a nuclear protein extraction kit (P0027, Beyotime Biotechnology, Shanghai, China). A 50 bp fluorescently labeled wild-type probe was designed from the goat *ENO1* promoter sequence region. In addition, unlabeled wild-type and mutated probes were produced. All probes were purchased from Sangon Biotech Co., Shanghai, China. The sequence of the fluorescently labeled and unlabeled wild-type probes is: F: 5′-CATGCGTGCGGGACTCGGAGTACGTGACGGAACCCCGAGTCCTCAT-3′, and the mutated probe is: F: 5′-CATGCGTGCGGG-ACTCGGAGTGTACGACGGAACCCCGAGTCCTCAT-3′. EMSA was performed using EMSA/Gel-Shift binding buffer (5×) according to the manufacturer’s instructions (GS005, Beyotime Biotechnology). A total of 0.08 pmol of fluorescently labeled wild-type probe was added to each reaction, and a 100-fold excess of unlabeled wild-type or mutated probe was added if competition analysis was to be performed. Three micrograms of nuclear-extracted protein containing XBP1s was used for each reaction. In the case of antibody addition, 0.5 μg of XBP1s antibody was used. Nucleoprotein complexes were separated by electrophoresis on 6% polyacrylamide gels and were visualized using a gel imaging system (GBox-Chemi-XRQ, Syngene, Cambridge, UK).

### 2.8. Glycogen Periodic Acid-Schiff Staining

A periodic acid-Schiff (PAS) staining kit (C0142, Beyotime Biotechnology) was used for detecting glycogen according to the procedure provided by the manufacturer. Briefly, cells were fixed, and periodic acid solution was added dropwise. The reaction was performed in a wet box protected from light for 10 min. The sample was washed, Schiff’s reagent was added dropwise, and the sample was placed in a wet box in an oven at 37 °C, protected from light, for 30 min. The sample was washed, hematoxylin staining solution was added dropwise, and the staining was continued for 30 s. The sample was washed, and the slide was sealed with sealing solution and photographed under a microscope (Nikon, Tokyo, Japan).

### 2.9. Data Statistics and Analysis

Data are expressed as the mean ± SEM, unless otherwise stated. When two groups were compared, statistical significance was determined using Student’s *t*-test. For comparisons involving more than two groups, one-way ANOVA was performed, followed by Tukey’s post hoc test for multiple comparisons. All statistical analyses were performed using GraphPad Prism version 8.0.1 (GraphPad Software Inc., San Diego, CA, USA). Differences were considered statistically significant when *p* < 0.05. At least three independent replicates were performed for each experiment.

## 3. Results

### 3.1. IFN-τ Inhibits Glycolysis and Lactate Production in Peri-Implantation gEECs

**Figure 1:**
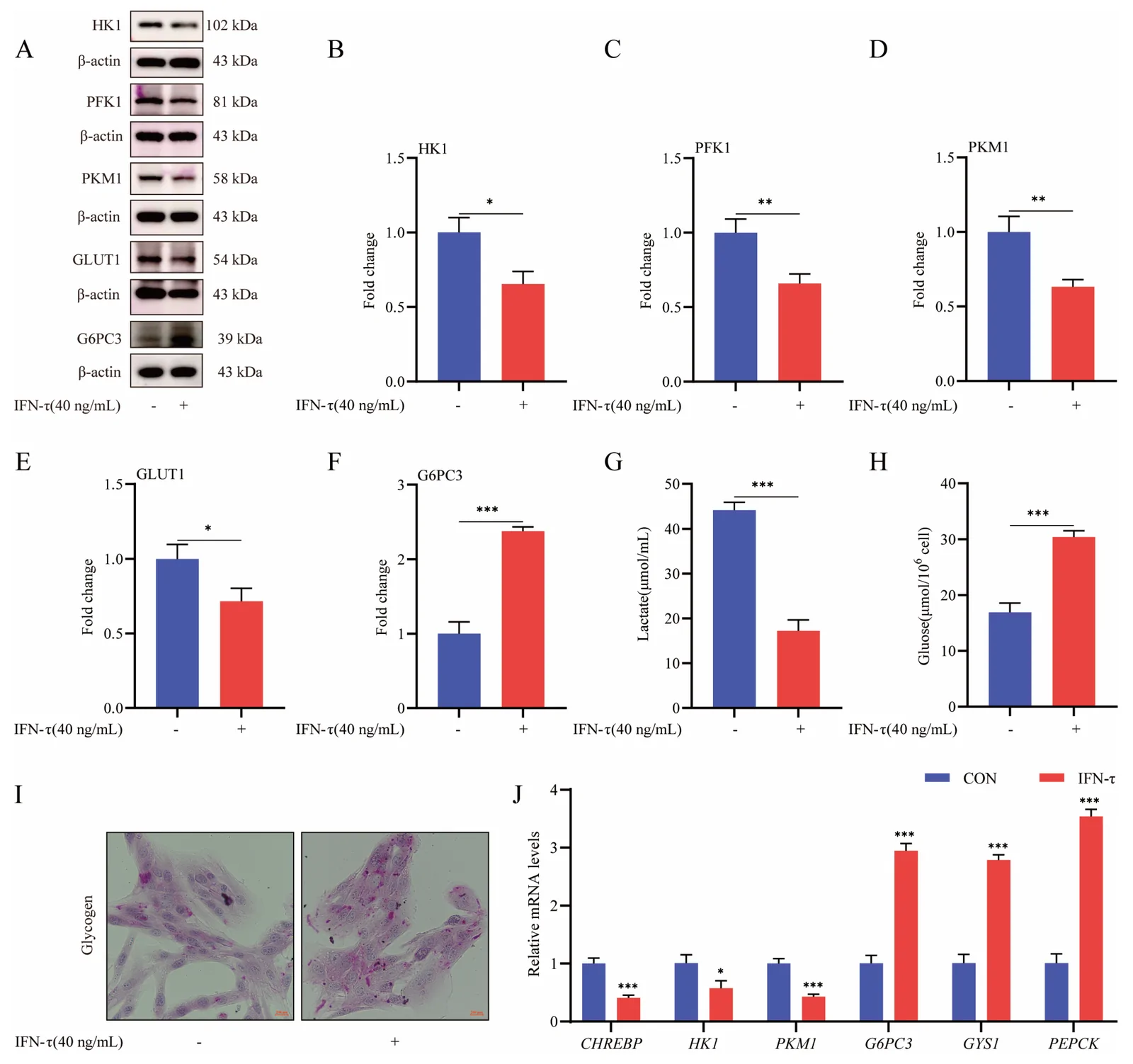
IFN-τ inhibits lactate production in gEECs. (**A**) Western blotting of HK1, PFK1, PKM1, GLUT1, and G6PC3 after IFN-τ treatment. Levels of β-actin are shown as the loading control. (**B**–**F**) Densitometric analysis of Western blotting of HK1, PFK1, PKM1, GLUT1, and G6PC3 after IFN-τ treatment, shown in panel A. (**G**) Lactate production in gEECs after IFN-τ treatment. (**H**) Glycogen content of gEECs after IFN-τ treatment. (**I**) Glycogen periodic acid-Schiff staining of gEECs after IFN-τ treatment. (**J**) Relative mRNA expression of *CHREBP*, *HK1*, *PKM1*, *G6PC3*, *GYS1*, and *PEPCK* was normalized to the level of *β-actin* as quantified by RT-qPCR. Data values are means ± SEM of three independent experiments. * *p* < 0.05; ** *p* < 0.01; *** *p* < 0.001.

To investigate the glycolytic changes in endometrial epithelial cells of ruminants during the peri-implantation period, gEECs were treated with IFN-τ, followed by analysis of the expression of glycolysis rate-limiting enzymes, including HK1, PFK-1, and PKM1, as well as by monitoring lactate production. IFN-τ treatment inhibited (*p* < 0.01) production of HK1, PFK-1, and PKM1, as well as lactate (Figure 1A–D,G). Production of the glucose transporter GLUT1 also decreased significantly (*p* < 0.05; Figure 1A,E), whereas the glycogen synthesis-related protein G6PC3 and glycogen content increased (*p* < 0.05; Figure 1A,F,H,I). Consistently, mRNA levels of the glycolysis-related genes *CHREBP*, *HK1*, and *PKM1* decreased, and expression of glycogen synthesis-related genes *G6PC3*, *GYS1*, and *PEPCK* increased in gEECs treated with IFN-τ compared with the controls (*p* < 0.001; Figure 1J). In summary, these results suggest that IFN-τ administration inhibits glycolysis in gEECs.

### 3.2. Overexpression of XBP1s Promotes Glycolysis and Lactate Production in Peri-Implantation gEECs

**Figure 2:**
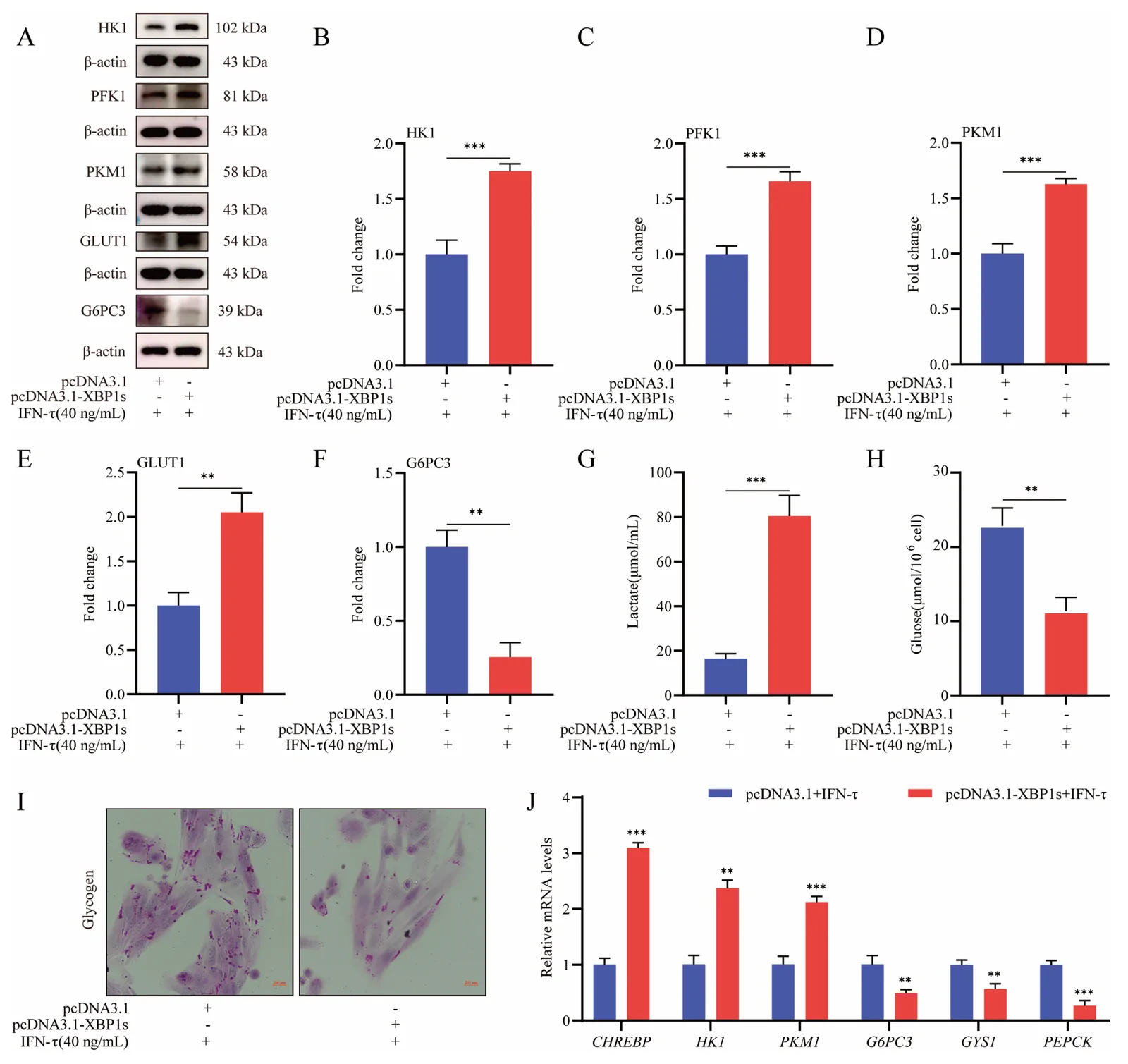
*XBP1s* overexpression promotes lactate production in gEECs. (**A**) Western blotting results of HK1, PFK1, PKM1, GLUT1, and G6PC3 after overexpression of *XBP1s*. Levels of β-actin are shown as the loading control. (**B**–**F**) Densitometric analysis of Western blotting of HK1, PFK1, PKM1, GLUT1, and G6PC3 after overexpression of *XBP1s* is shown in panel A. (**G**) Lactate production in gEECs after overexpression of *XBP1s*. (**H**) Glycogen content of gEECs following overexpression of *XBP1s*. (**I**) Glycogen periodic acid-Schiff staining of gEECs following overexpression of *XBP1s*. (**J**) The mRNA levels of *CHREBP*, *HK1*, *PKM1*, *G6PC3*, *GYS1*, and *PEPCK* after overexpression of *XBP1s* were normalized to the level of *β-actin* as quantified by RT-qPCR. Data values are means ± SEM of three independent experiments. ** *p* < 0.01; *** *p* < 0.001.

**Figure 3:**
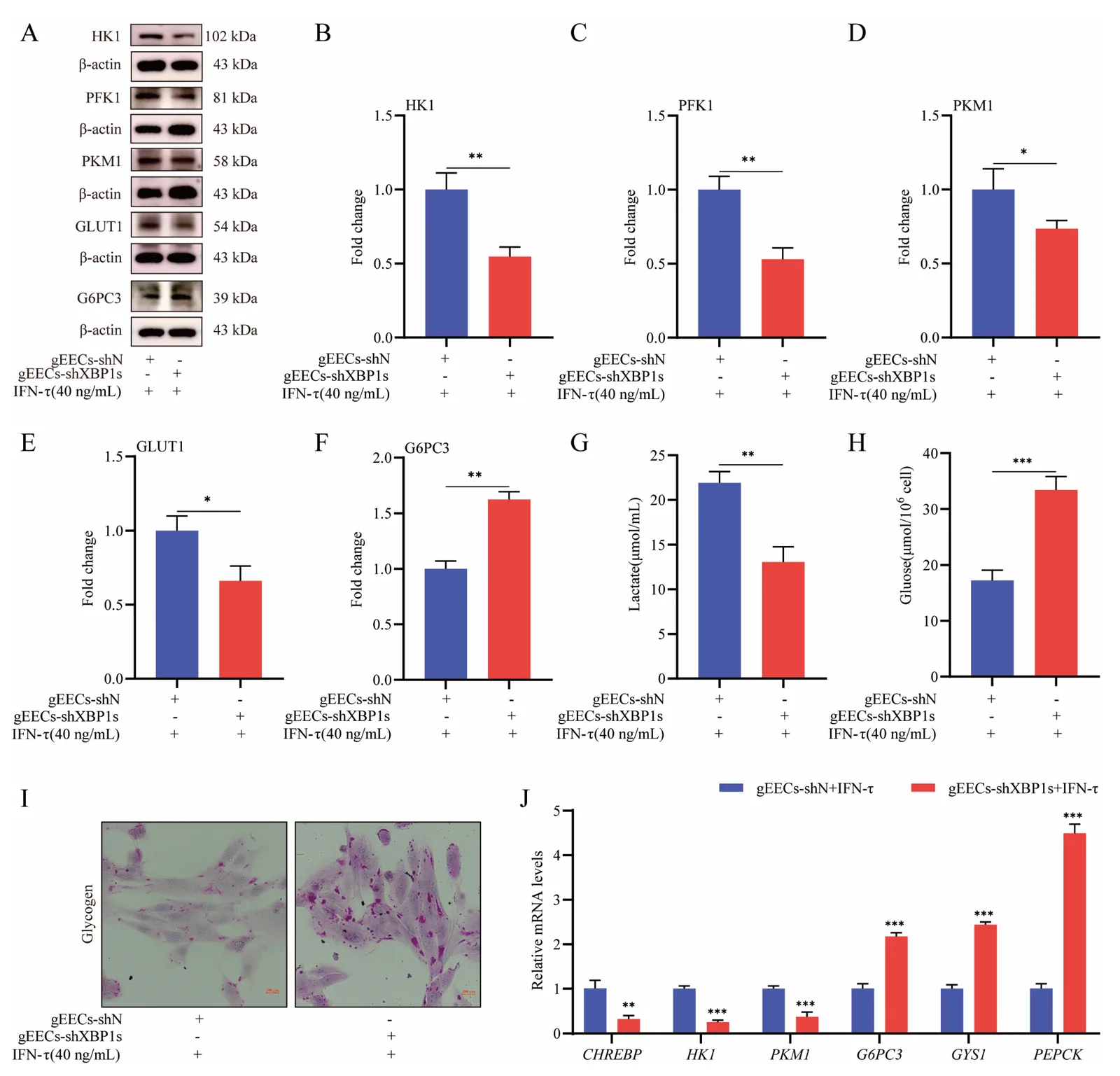
*XBP1s* knockdown inhibits glycolysis in gEECs. (**A**) Relative protein levels of HK1, PFK1, PKM1, GLUT1, and G6PC3 assessed by Western blotting after knockdown of *XBP1s*. Levels of β-actin are shown as the loading control. (**B**–**F**) Densitometric analysis of Western blotting of HK1, PFK1, PKM1, GLUT1, and G6PC3 after overexpression of *XBP1s* is shown in panel A. (**G**) Lactate production in gEECs after knockdown of *XBP1s*. (**H**) Glycogen content of gEECs after knockdown of *XBP1s*. (**I**) Glycogen periodic acid-Schiff staining of gEECs after knockdown of *XBP1s*. (**J**) The mRNA levels of *CHREBP*, *HK1*, *PKM1*, *G6PC3*, *GYS1*, and *PEPCK* after knockdown of *XBP1s* were normalized to the level of *β-actin* as quantified by RT-qPCR. Data values are means ± SEM of three independent experiments. * *p* < 0.05; ** *p* < 0.01; *** *p* < 0.001.

A previous study has shown that transcription factor XBP1s was significantly suppressed during the formation of endometrial receptivity, and its expression pattern is synchronized with that of glycolytic markers during the implantation period [30]. In addition, our CUT&Tag experiments revealed that XBP1s targeted genes that were enriched for the glycolytic pathway [31]. Therefore, we hypothesized that the protein may regulate endometrial receptivity of gEECs by regulating glycolysis. The effect of XBP1s on glycolysis in peri-implantation gEECs was explored further by assessing the impact of overproduction of the protein on marker genes at both mRNA and protein levels. Production of the HK1, PFK-1, PKM1, and GLUT1 proteins was enhanced significantly (*p* < 0.01) after overexpression of *XBP1s* (Figure 2A–E). Lactate content also increased (*p* < 0.001) as a consequence of *XBP1s* overexpression compared with the control (Figure 2G). In contrast, the levels of the G6PC3 protein and glycogen content in gEECs decreased significantly (*p* < 0.01; Figure 2A,F,H,I). In addition, mRNA levels of *CHREBP*, *HK1*, and *PKM1* were elevated, whereas expression of the *G6PC3*, *GYS1*, and *PEPCK* genes decreased with *XBP1s* overexpression (*p* < 0.01; Figure 2J). Consistently, silencing of *XBP1s* inhibited the production of the HK1, PFK-1, PKM1, and GLUT1 proteins, reduced mRNA levels of the *CHREBP*, *HK1*, and *PKM1* genes, and suppressed lactate production (*p* < 0.01; Figure 3A–E,G,J). However, levels of the G6PC3 protein, mRNA expression of *G6PC3*, *GYS1*, and *PEPCK*, and glycogen synthesis were significantly enhanced (*p* < 0.01; Figure 3A,F,H–J). Thus, overexpression of *XBP1s* stimulates glycolysis and lactate production in peri-implantation gEECs.

### 3.3. XBP1s Promotes Transcription of the ENO1 Gene by Binding to the Promoter

**Figure 4:**
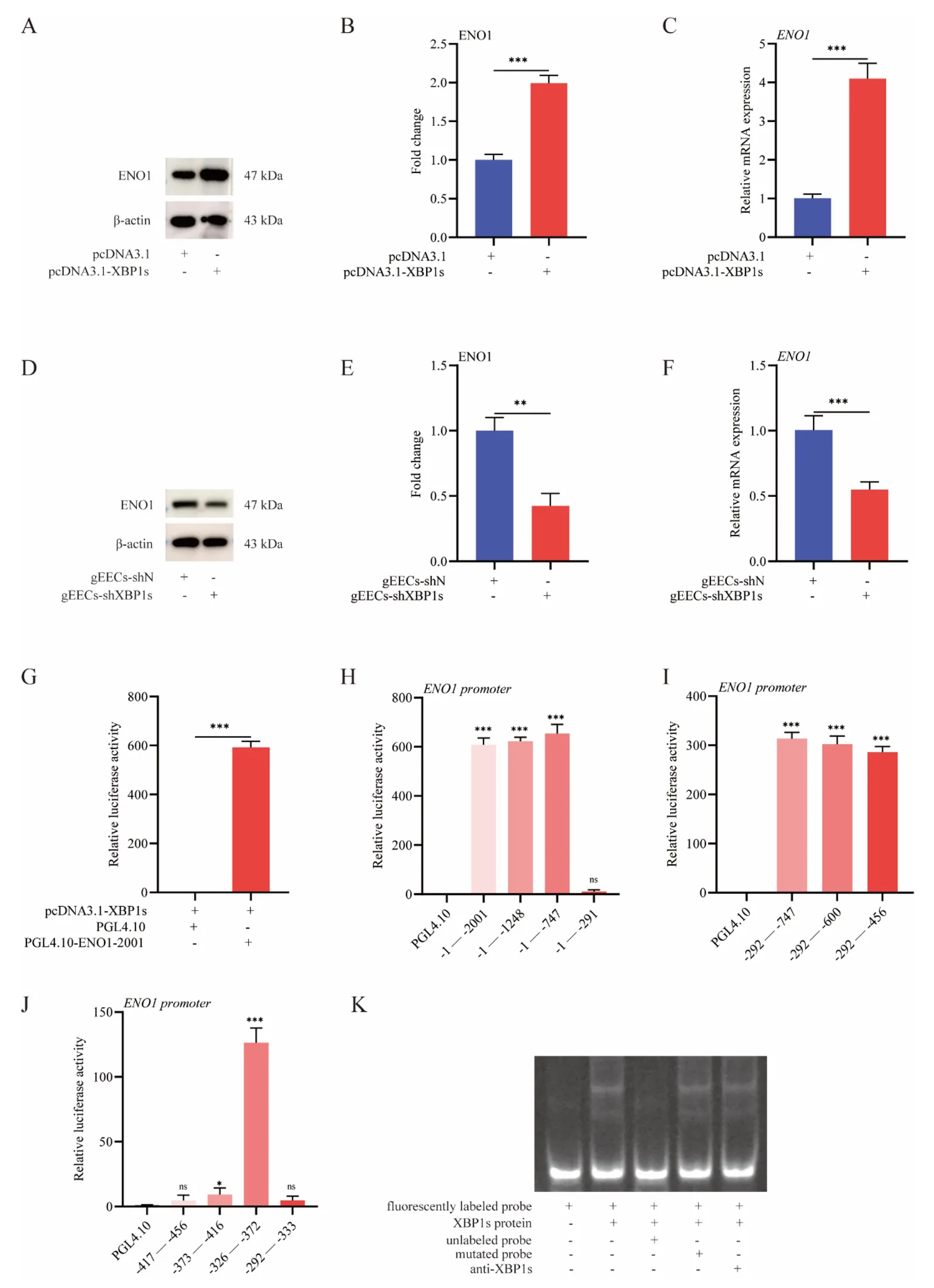
XBP1s promotes the transcriptional activity of the *ENO1* promoter. (**A**,**B**) Relative protein level of ENO1 assessed by Western blotting after overexpression of *XBP1s*. (**C**) The mRNA levels of *ENO1* after overexpression of *XBP1s*. (**D**,**E**) Relative protein level of ENO1 assessed by Western blotting after knockdown of *XBP1s*. (**F**) The mRNA levels of *ENO1* after knockdown of *XBP1s*. (**G**) Relative luciferase activities of PGL4.10-ENO1 and empty vector PGL4.10 were measured in HEK 293T cells that were co-transfected with pcDNA3.1-XBP1s. (**H**–**J**) Relative luciferase activities of PGL4.10-ENO1 constructs with the indicated deletions were measured in HEK 293T cells that were co-transfected with pcDNA3.1-XBP1s. (**K**) EMSA using fluorescently labeled wild-type, unlabeled wild-type, and unlabeled mutated probes was performed to measure the binding of XBP1s to the *ENO1* promoter. Data values are means ± SEM of three independent experiments. ^ns^ no significant difference, * *p* < 0.05; ** *p* < 0.01; *** *p* < 0.001.

The mechanism by which transcription factor XBP1s acts in glycolysis was explored further by analyzing our previous CUT&Tag results, which revealed that *ENO1*, which encodes the enzyme in the ninth step of glycolysis (ENO1 catalyzes the conversion of 2-phosphoglycerate to phosphoenolpyruvate during glycolysis), may serve as a target gene of transcription factor XBP1s [31]. Accordingly, mRNA and protein levels of ENO1 increased significantly after overexpression of *XBP1s* in gEECs, whereas silencing of *XBP1s* significantly suppressed *ENO1* expression (*p* < 0.01; Figure 4A–F). A dual-luciferase reporter plasmid driven by the *ENO1* promoter (PGL4.10-ENO1) and the pcDNA3.1-XBP1s expression plasmid were co-transfected into HEK 293T cells to assess further whether this regulation was mediated by the direct binding of XBP1s to the *ENO1* promoter. The transcriptional activity of *ENO1* increased significantly following co-transfection with pcDNA3.1-XBP1s (*p* < 0.001; Figure 4G). The binding site for XBP1s in the *ENO1* promoter was initially defined using truncated *ENO1* promoter sequences in a dual-luciferase reporter, which implicated the sequence from −372 to −326 as the binding site for XBP1s (Figure 4H–J). EMSAs were subsequently employed to further localize the XBP1s binding site within the *ENO1* promoter sequence. A distinct retarded nucleoprotein complex was detected when nuclear proteins extracted from gEECs containing XBP1s were incubated with a fluorescently labeled probe that encompassed the putative binding site within the *ENO1* promoter (−372 to −326). This species was absent when an unlabeled probe was used as a competitor in the binding reaction, but reappeared in the presence of a mutated competitor sequence. In addition, the intensity of the retarded complex decreased when XBP1s antibody was added to the reaction, which verified that the protein within the complex was XBP1s (Figure 4K). The combined in vivo and in vitro data indicate that XBP1s binds directly to the *ENO1* promoter to positively regulate transcriptional activity of *ENO1*.

### 3.4. ENO1 Inhibits Endometrial Receptivity

**Figure 5:**
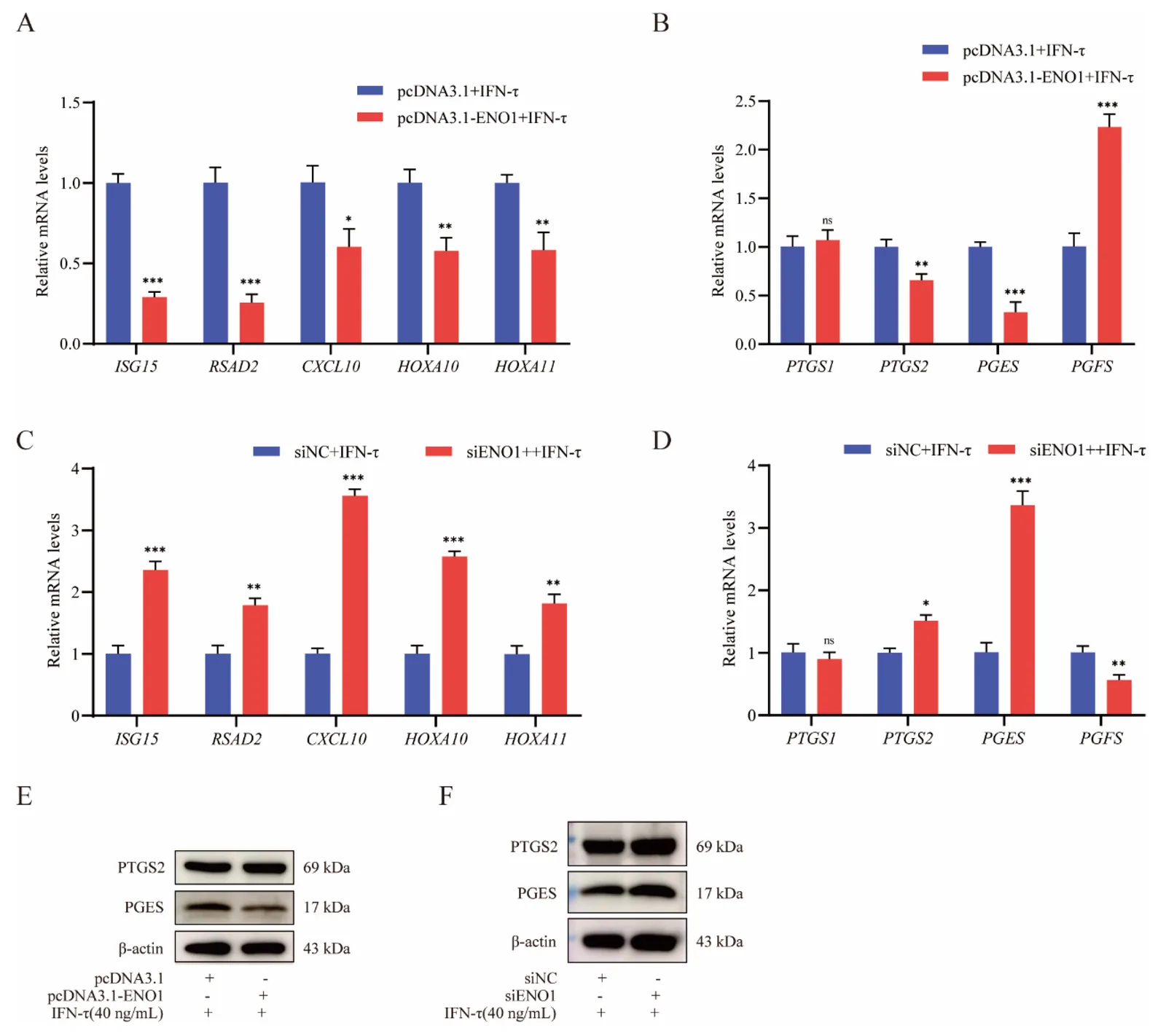
Effect of *ENO1* on endometrial receptivity. (**A**) Relative mRNA levels of *ISG15*, *RSAD2*, *CXCL10*, *HOXA10*, and *HOXA11* after overexpression of *ENO1*. (**B**) The mRNA levels of *PTGS1*, *PTGS2*, *PGES*, and *PGFS* after overexpression of *ENO1*. (**C**) Relative mRNA levels of *ISG15*, *RSAD2*, *CXCL10*, *HOXA10*, and *HOXA11* after knockdown of *ENO1*. (**D**) The mRNA levels of *PTGS1*, *PTGS2*, *PGES*, and *PGFS* after knockdown of *ENO1*. (**E**) Relative protein levels of *PTGS2* and *PGES* assessed by Western blotting after overexpression of *ENO1*. (**F**) Relative protein levels of *PTGS2* and *PGES* assessed by Western blotting after knockdown of *ENO1*. Data values are means ± SEM of three independent experiments. ^ns^ no significant difference, * *p* < 0.05; ** *p* < 0.01; *** *p* < 0.001.

In view of the role of XBP1s in regulation of the *ENO1* gene, we explored the contribution of ENO1 to endometrial receptivity. Overproduction of ENO1 in gEECs inhibited expression of the *ISG15*, *RSAD2*, *CXCL10*, *HOXA10*, and *HOXA11* marker genes, whereas knockdown of *ENO1* promoted endometrial receptivity (*p* < 0.01; Figure 5A,C; Figure S1A–F). In agreement, *PGES* mRNA and protein levels were suppressed by overexpression of *ENO1*, whereas *PGFS* expression was increased. Knockdown of *ENO1* exerted the converse effect (*p* < 0.01; Figure 5B,D–F). These combined data suggest that ENO1 hinders endometrial receptivity in gEECs.

### 3.5. ENO1 Promotes Glycolysis and Lactate Production in Peri-Implantation gEECs

**Figure 6:**
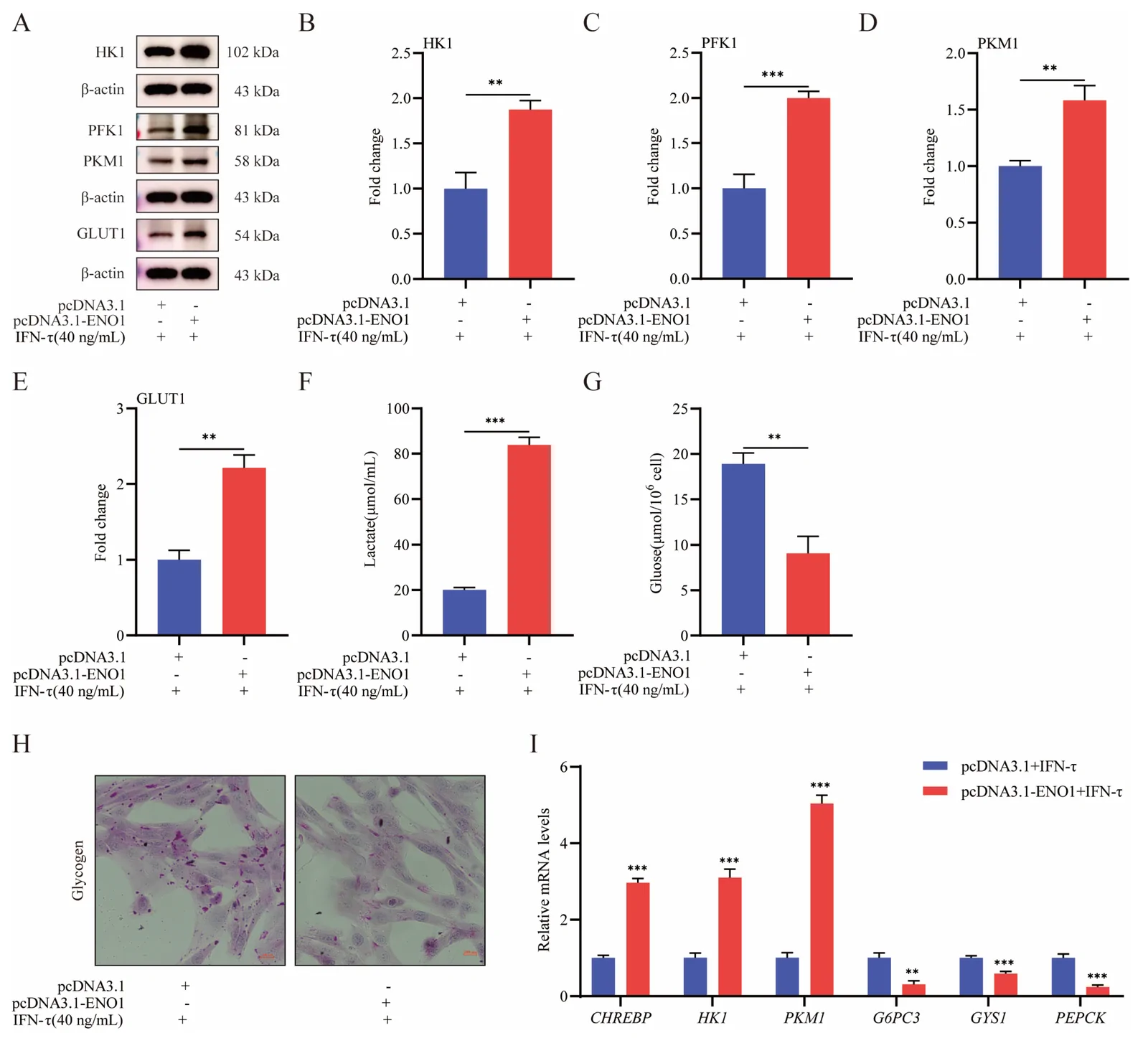
Overexpression of *ENO1* promotes glycolysis in gEECs. (**A**) Relative protein levels of HK1, PFK1, PKM1, and GLUT1 assessed by Western blotting after overexpression of *ENO1*. Levels of β-actin are shown as the loading control. (**B**–**E**) Densitometric analysis of Western blotting of HK1, PFK1, PKM1, and GLUT1 after overexpression of *XBP1s* is shown in panel A. (**F**) Determination of lactate content after overexpression of *ENO1*. (**G**) Determination of glycogen content in gEECs after overexpression of *ENO1*. (**H**) Glycogen periodic acid-Schiff staining in gEECs after overexpression of *ENO1*. (**I**) Relative mRNA levels of *CHREBP*, *HK1*, *PKM1*, *G6PC3*, *GYS1*, and *PEPCK* after overexpression of *ENO1* were normalized to the level of *β-actin* as quantified by RT-qPCR. Data values are means ± SEM of three independent experiments. ** *p* < 0.01; *** *p* < 0.001.

**Figure 7:**
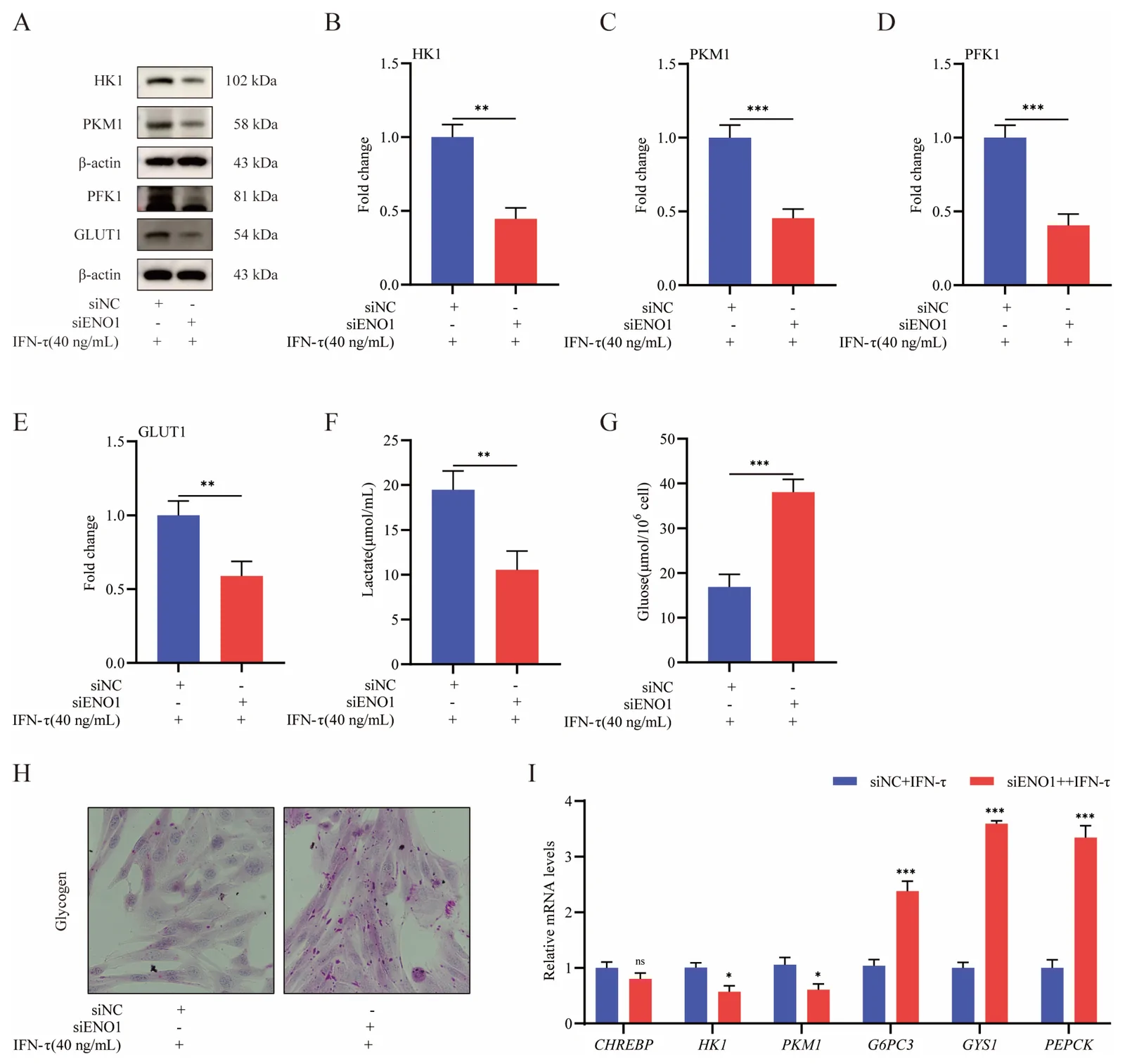
Knockdown of *ENO1* inhibits lactate production in gEECs. (**A**) Relative protein levels of HK1, PFK1, PKM1, and GLUT1 assessed by Western blotting after knockdown of *ENO1*. Levels of β-actin are shown as the loading control. (**B**–**E**) Densitometric analysis of Western blotting of HK1, PFK1, PKM1, and GLUT1 after knockdown of *ENO1* is shown in panel A. (**F**) Determination of lactate content after knockdown of *ENO1*. (**G**) Determination of glycogen content after knockdown of *ENO1*. (**H**) Glycogen periodic acid-Schiff staining in gEECs after knockdown of *ENO1*. (**I**) Relative mRNA levels of *CHREBP*, *HK1*, *PKM1*, *G6PC3*, *GYS1*, and *PEPCK* after knockdown of *ENO1* were normalized to the level of *β-actin* as quantified by RT-qPCR. Data values are means ± SEM of three independent experiments. ^ns^ no significant difference, * *p* < 0.05; ** *p* < 0.01; *** *p* < 0.001.

The role of ENO1 in glycolysis and lactate production in peri-implantation gEECs was examined by overexpressing and knocking down the gene and assessing the impact on expression of marker genes for glycolysis, as well as on lactate and glycogen concentrations. Overexpression of *ENO1* significantly promoted HK1, PFK-1, PKM1, and GLUT1 protein levels (*p* < 0.01; Figure 6A–E), and in parallel, increased the lactate content (*p* < 0.001; Figure 6F). However, the glycogen content of gEECs was suppressed by *ENO1* overexpression (*p* < 0.01; Figure 6G,H). In addition, mRNA levels of the *CHREBP*, *HK1*, and *PKM1* genes increased appreciably, whereas expression of *GYS1*, *G6PC3*, and *PEPCK* decreased during *ENO1* overexpression (*p* < 0.01; Figure 6I). Consistently, knockdown of *ENO1* in gEECs reduced HK1, PFK-1, PKM1, and GLUT1 protein levels as well as expression of the *CHREBP*, *HK1*, and *PKM1* genes. Lactate production was also suppressed (*p* < 0.01; Figure 7A–F,I). In contrast, the knockdown promoted expression of the *G6PC3*, *GYS1*, and *PEPCK* genes, and stimulated glycogen synthesis (*p* < 0.01; Figure 7G–I). Thus, these overexpression and knockdown data strongly suggest that ENO1 enhances glycolysis and lactate production in peri-implantation gEECs.

### 3.6. Lactate Inhibits Endometrial Receptivity of gEECs

**Figure 8:**
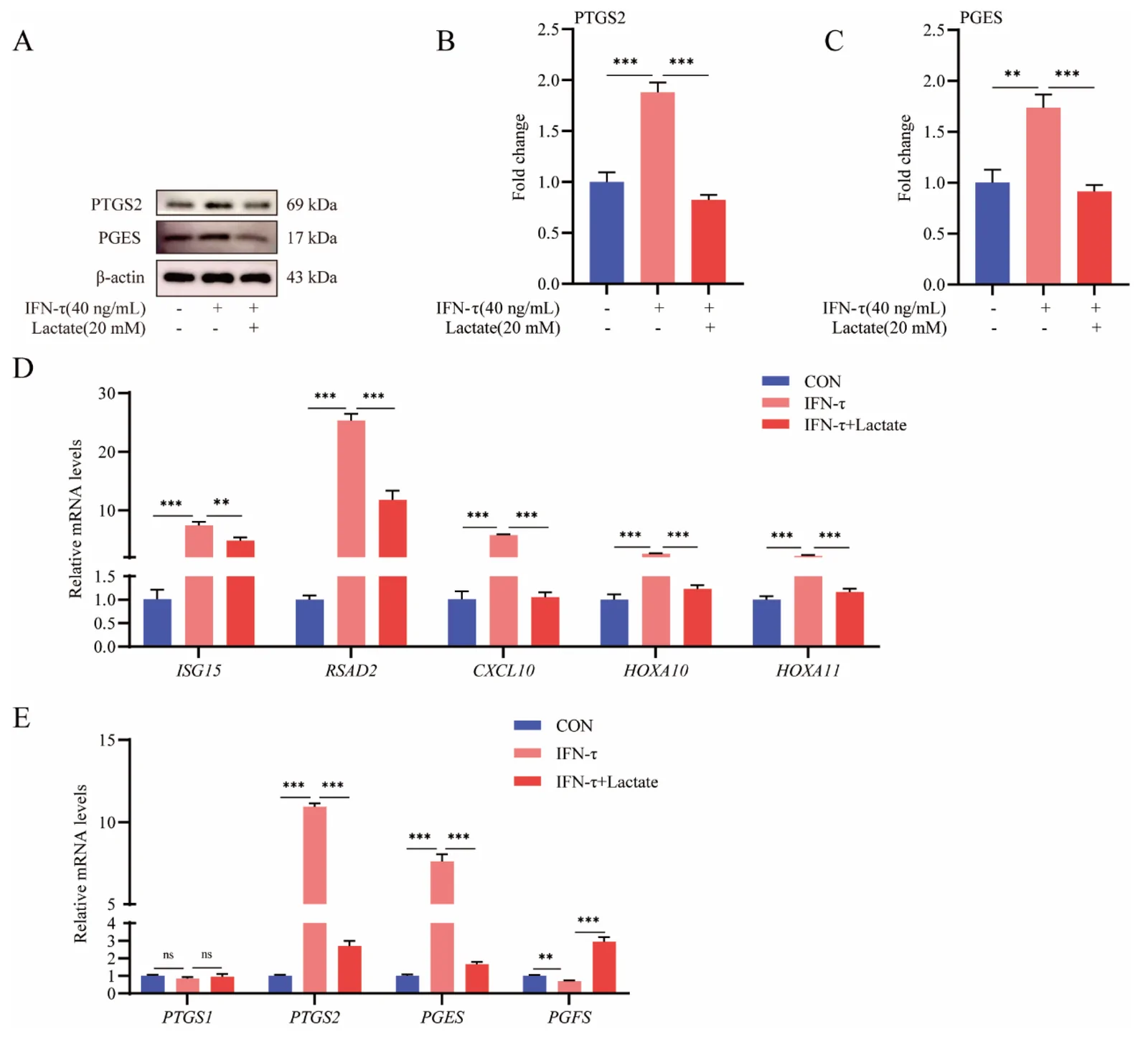
Lactate inhibits IFN-τ-induced endometrial receptivity in gEECs. (**A**) Relative protein levels of *PTGS2* and *PGES*, assessed by Western blotting after treatment with lactate. Levels of β-actin are shown as the loading control. (**B**,**C**) Densitometric analysis of Western blotting of *PTGS2* and *PGES* after treatment with lactate is shown in panel A. (**D**) Relative mRNA levels of *ISG15*, *RSAD2*, *CXCL10*, *HOXA10*, and *HOXA11* after treatment with lactate. (**E**) Relative mRNA levels of *PTGS1*, *PTGS2*, *PGES*, and *PGFS* after treatment with lactate. Data values are means ± SEM of three independent experiments. ^ns^ no significant difference, ** *p* < 0.01; *** *p* < 0.001.

Lactate, the end product of glycolysis, is involved in diverse biochemical and physiological processes. Since the preceding data suggest that IFN-τ inhibits lactate production during the peri-implantation period, we next explored the effect of lactate on IFN-τ-induced endometrial receptivity in gEECs. Exogenous lactate (20 mM) significantly inhibited expression of the *ISG15*, *RSAD2*, *CXCL10*, *HOXA10*, and *HOXA11* genes (*p* < 0.01; Figure 8D). In addition, production of the PTGS2 and PGES proteins decreased in lactate-treated gEECs compared with cells that were treated only with IFN-τ (*p* < 0.001; Figure 8A–C). Expression of the *PTGS2* and *PGES* loci also decreased significantly, whereas expression of *PGFS* increased (*p* < 0.001; Figure 8E). These results indicate that lactate inhibits IFN-τ-induced endometrial receptivity in gEECs.

## 4. Discussion

Endometrial receptivity comprises a complex set of events at both molecular and cellular levels during a defined period of early pregnancy. The process is critical for successful embryo implantation and pregnancy establishment. Glycolysis is one of the most fundamental pathways in cellular energy metabolism, the conversion of glucose to pyruvate, and then to lactate by a series of enzymes [32]. Lactate is involved as a signaling molecule in the regulation of cellular metabolism, promoting glycolysis and enhancing cellular adaptation to the environment [33]. However, the effect of lactate on endometrial function in ruminants remains unclear. Therefore, we explored glycolytic changes in IFN-τ-induced endometrial receptivity in these cells, which revealed that IFN-τ inhibited expression of the glycolysis rate-limiting enzymes HK1, PFK1, and PKM1 at mRNA and protein levels, reduced expression of *GLUT1*, and also inhibited production of the glycolytic end product lactate. However, expression of the glycogen synthesis-associated genes *G6PC3*, *PEPCK*, and *GYS1*, and glycogen content increased following IFN-τ treatment. These combined results suggest that IFN-τ inhibits glycolysis in gEECs. In agreement, IFN-I impeded glycolysis in inflammatory macrophages and initiated mitochondrial stress [34], and also repressed glycolysis in Vero cell cultures after *Usutu* virus infection [35]. The ERS pathway is also involved in the formation of endometrial receptivity, but the connections between the processes have not yet been clarified. The XBP1s protein is a key transcription factor in the ERS pathway and was hypothesized to be involved in the regulation of endometrial receptivity [36]. Since our CUT&Tag experiments revealed that XBP1s targets genes that are enriched for the glycolytic pathway [31], we hypothesized that XBP1s may regulate endometrial receptivity by modulating glycolysis. Exploring the effects of XBP1s on glycolysis during the establishment of endometrial receptivity revealed that overexpression of the protein enhanced expression of HK1, PFK1, PKM1, and GLUT1 at both mRNA and protein levels, stimulated lactate production, and reversed the effects of IFN-τ on glycolysis. Conversely, silencing of *XBP1s* enhanced the effects of IFN-τ on glycolysis. Previous studies have generated analogous results. For example, XBP1s enhanced glycolysis in classically activated macrophages that were subjected to lipopolysaccharide stimulation or that were infected with the intracellular bacterial pathogen *Brucella abortus* [37]. In short, we determined that IFN-τ promotes endometrial receptivity in gEECs by suppressing glycolysis, whereas XBP1s reverses this effect by enhancing glycolysis and lactate production, thereby inhibiting the establishment of endometrial receptivity.

ENO1 catalyzes the conversion of 2-phosphoglycerate to phosphoenolpyruvate during glycolysis. Analysis of our CUT&Tag sequencing data suggested that the enzyme might be a target gene for transcriptional control by XBP1s. In agreement, dual-luciferase reporter assays showed that XBP1s stimulated the activity of the *ENO1* promoter sequence (−2001 to −1 bp). We truncated the promoter and determined that the region from −372 to −326 serves as the regulatory binding site for XBP1s based on additional reporter and EMSA assays. These observations led us to probe the effects of ENO1 on endometrial receptivity and glycolysis. Overexpression of the enzyme inhibited IFN-τ-induced expression of *ISG15*, *RSAD2*, *CXCL10*, *HOXA10*, *HOXA11*, and *PGES*, stimulated expression of *PGFS*, and reduced IFN-τ-induced establishment of endometrial receptivity, whereas the *ENO1* knockdown ameliorated establishment of endometrial receptivity. These observations parallel our findings that implicate XBP1s in endometrial receptivity. In addition, overexpression of *ENO1* promoted the expression of *HK1*, *PFK1*, *PKM1*, and *GLUT1*, enhanced lactate production, and reversed the effect of IFN-τ on glycolysis, whereas knockdown of the gene instead produced the converse effects and enhanced the impact of IFN-τ on glycolysis. These combined results confirm our hypothesis that XBP1s promotes *ENO1* transcription by directly binding to the *ENO1* promoter, which, in turn, boosts glycolysis and lactate production in gEECs. Similarly, *ENO1* and *XBP1s* show comparable expression patterns during kaempferol-induced neuroblastoma differentiation [38].

Glycolysis involves a stepwise series of enzymatic reactions that ultimately convert glucose to lactate. Lactate plays an essential role as a bridging signaling molecule that coordinates interactions between diverse cells, organs, and tissues [39]. Since we found that IFN-τ inhibited glycolysis and lactate production in peri-implantation gEECs, we determined the effect of lactate on IFN-τ-induced endometrial receptivity in these cells. IFN-τ-induced expression of endometrial receptivity marker genes was suppressed following pretreatment with exogenous lactate. Furthermore, expression of *PTGS2* and *PGES* decreased and expression of *PGFS* increased in lactate-treated gEECs, which suggests that lactate adversely affects the establishment of IFN-τ-induced endometrial receptivity. This conclusion is consistent with previous studies that showed that increased placental glycolysis and lactate production were detrimental to embryo implantation [40]. In addition, lactate is unfavorable for embryo elongation and development [41].

## 5. Conclusions

**Figure 9:**
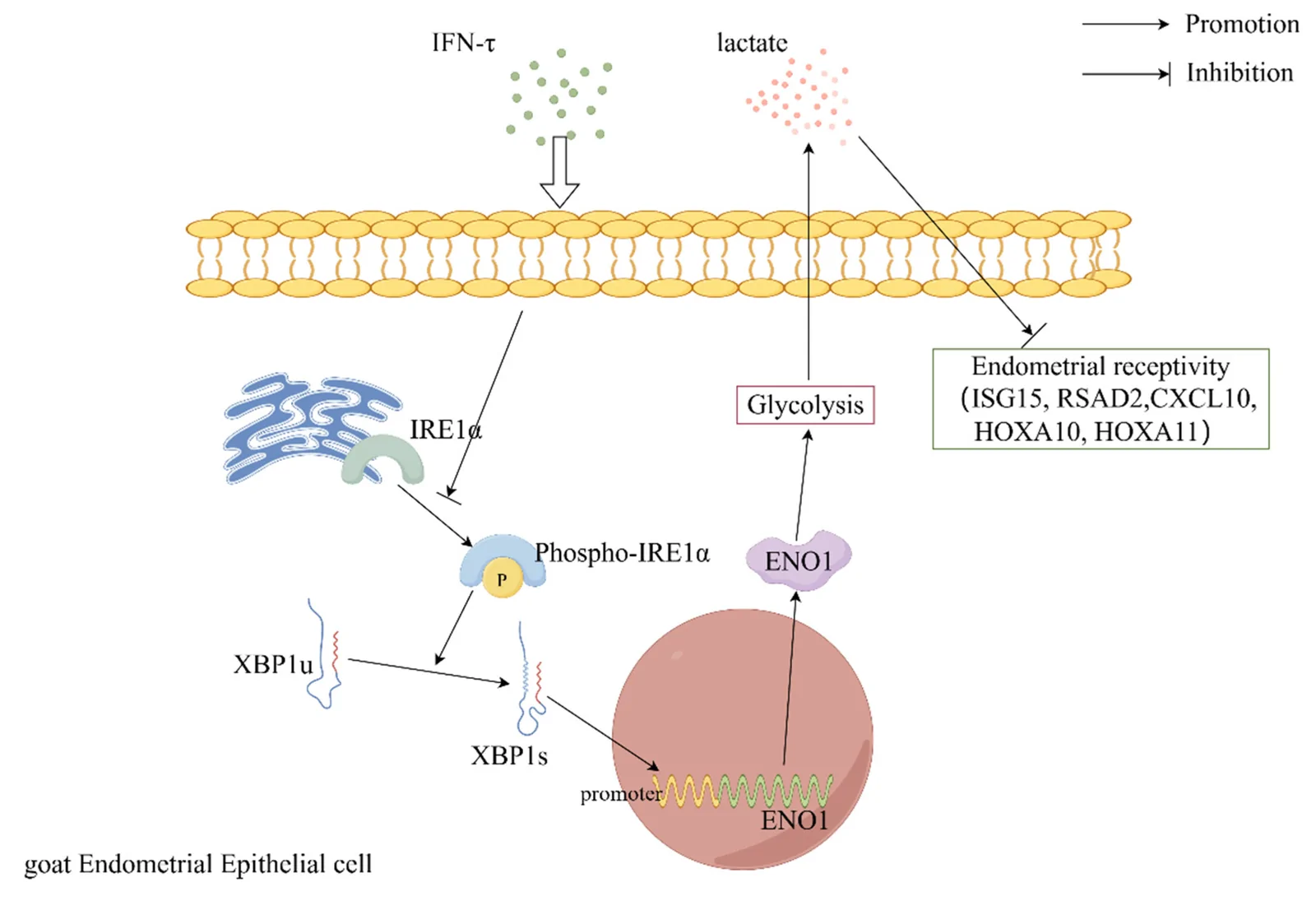
XBP1s inhibits IFN-τ-induced endometrial receptivity by promoting glycolysis and lactate production.

In conclusion, our results elucidated the mechanism by which the transcription factor XBP1s regulates IFN-τ-induced endometrial receptivity. The protein directly binds the promoter sequence of the glycolytic enzyme ENO1 and stimulates the transcriptional activity of *ENO1* to enhance glycolysis and lactate production. In contrast, increased lactate concentrations inhibit the establishment of IFN-τ-induced endometrial receptivity (Figure 9). The data provided a basis for further studies that address the problems of low reproduction and high culling rates in livestock farming.

## Figures and Tables

**Table 1:** Primer sequences used for RT-qPCR and construction of XBP1s overexpression vectors.

Target Gene	GenBank Accession No.	Primer Sequence 5′-3′
Used for q-PCR:		
*β-actin*	NM_001314342.1	F: CTTCCAGCCTTCCTTCCTGG
		R: GCCAGGGCAGTGATCTCTTT
*ISG15*	XM_005690795.3	F: GGTGAGGAACGACAAGGGTC
		R: CAGAATTGGTCCGCTTGCAC
*RSAD2*	XM_018055702.1	F: TGCTTGGTGCCCGAGTCTAAC
		R: TCCGCCCATTTCTACAGTTCA
*CXCL10*	NM_001285721.1	F: AGGAATACACGCTGTACCTGC
		R: ACGTGGGCAGGATTGACTTG
*HOXA10*	XM_018047091.1	F: CTTCCAAAGGCGAAAACGCA
		R: GTCTGGTGCTTGGTGTAGGG
*XBP1s*		F: CCTGCCTGCTGGATGCTTA
		R: GGGAAAGAGTTCACTGGCAAA
*HOXA11*	XM_018047092.1	F: CAGATTCGGGAGCTAGAGCG
		R: CGGTCAGTGAGGTTGAGCAT
*PTGS1*	XM_005687044.3	F: GCGGCTCTTTAAGGATGGGA
		R: ATCGTGGCGTAGAGCATGAG
*PTGS2*	XM_018060731.1	F: TGTATCCCGCCCTTCTGGTA
		R: GAATGGTGCTCCGGCTTCTA
*PGES*	XM_018055956.1	F: GAGCTAATGAACGGCCAGGT
		R: TCGTTCCGGCAATACTGGAG
*PGFS*	XM_005688135.3	F: TGGAGGACCCAGTTCTTTGTG
		R: TACCTGATAGCGAAGGGCAAC
*CHREBP*	XM_018040995.1	F: TTCTTCACCAACTACCGCCC
		R: CGGTTATGCCCTGAGAGGTG
*PEPCK*	XM_005688314.3	F: CTCCTCTGTCCAAGATCGGC
		R: GGGTTACAGGCCCAGTTGTT
*G6PC3*	XM_005693916.3	F: CCTGGTTGGAGAACGTGTGG
		R: CTGATCCAGAGCACCGCAAT
*HK1*	XM_018042326.1	F: TCATTTCGCTCTGCCAACCT
		R: GGGTGCGTCTTGTAAAGGGA
*GYS1*	XM_018062746.1	F: GGCTACCATCTTCACCACCC
		R: ATGGCGGTAATCTGGGACAC
*PKM1*	XM_005685176.3	F: TGTCACCCATTACCAGCGAC
		R: CTGTCTGGTGATTCCGGGTC
Used for constructing a recombinant vector:		
*ENO1*	XM_018060394.1	F: ATGTCCATCCTGAAGGTCCATGC
		R: TTACTTGGCGAGCGGGTTTCTG
Used for constructing luciferase vector:		
ENO1-promoter		F: TTGGCAGGGCATTTGGCAGCCTG
		R: CGCCCACTTCCCGCCCCC

**Table 2:** siRNA sequence.

siRNA Name	Sequences (5′-3′)
siENO1-1	GUCGAGGUUGAUCUCUUCATT
	UGAAGAGAUCAACCUCGACTT
siENO1-2	GACAAGCUGAUGAUAGAGATT
	UCUCUAUCAUCAGCUUGUCTT
siENO1-3	GAUGACCCCAACAGGUACATT
	UGUACCUGUUGGGGUCAUCTT
siNC	UUCUCCGAACGUGUCACGUTT
	ACGUGACACGUUCGGAGAATT

## Data Availability

The datasets produced and analyzed during the current study are available from the corresponding author upon reasonable request.

## Supplementary Materials

The following supporting information can be downloaded at: https://www.mdpi.com/article/10.3390/cells15181656/s1, Figure S1: *ENO1* was successfully overexpressed and knocked down in gEECs. (A–C) Validation of ENO1 overexpression efficiency by Western blotting and RT-qPCR. (D–F) Validation of ENO1 knockdown efficiency by Western blotting and RT-qPCR. Data values are means ± SEM of three independent experiments. * *P* < 0.05; ** *P* < 0.01; *** *P* < 0.001.

## Abbreviations

XBP1s, X-box binding protein 1 splicing variant; ENO1, enolase 1; IFN-τ, interferon-tau; E_2_, estrogen; P_4_, progesterone; PGES, prostaglandin E2 synthase; PGFS, prostaglandin F2-alpha synthase; ISG15, interferon-stimulated gene 15; CXCL10, chemokine (C-X-C motif) ligand 10; RSAD2, s-adenosyl domain-containing protein 2; PTGS1, prostaglandin-endoperoxide synthase 1; PTGS2, prostaglandin-endoperoxide synthase 2; HOXA10, homeobox A10; HOXA10, homeobox A11; HK1, hexokinase 1; PFK-1, phosphofructokinase-1; PKM1, pyruvate kinase M1; GLUT1, glucose transporter type 1; G6PC3, glucose-6-phosphatase catalytic subunit 3; CHREBP, carbohydrate response element binding protein; GYS1, glycogen synthase 1; PEPCK, phosphoenolpyruvate carboxy kinase; EMSA, electrophoretic mobility shift assay; ERS, endoplasmic reticulum stress; gEECs, goat endometrial epithelial cells; UPR, unfolded protein response; IRE1α, inositol-requiring enzyme 1α; GRP78, glucose regulated protein 78; PBS, phosphate-buffered saline; hTERT, human telomerase reverse transcriptase; DF12, Dulbecco’s modified Eagle’s medium (DMEM)/F-12; DMEM, Dulbecco’s modified Eagle’s medium; FBS, fetal bovine serum; SDS-PAGE, sodium dodecyl sulfate polyacrylamide gel electrophoresis.

## References

[1] Matsuno Y., Imakawa K. (2025). Biological Aging and Uterine Fibrosis in Cattle: Reproductive Trade-Offs from Enhanced Productivity. Cells.

[2] Davenport K.M., Ortega M.S., Johnson G.A., Seo H., Spencer T.E. (2023). Review: Implantation and placentation in ruminants. Animal.

[3] Spencer T.E., Hansen T.R. (2015). Implantation and Establishment of Pregnancy in Ruminants. Adv. Anat. Embryol. Cell Biol..

[4] Forde N., Lonergan P. (2017). Interferon-tau and fertility in ruminants. Reproduction.

[5] Rocha C.C., da Silveira J.C., Forde N., Binelli M., Pugliesi G. (2021). Conceptus-modulated innate immune function during early pregnancy in ruminants: A review. Anim. Reprod..

[6] Lee J., McCracken J.A., Stanley J.A., Nithy T.K., Banu S.K., Arosh J.A. (2012). Intraluteal prostaglandin biosynthesis and signaling are selectively directed towards PGF2alpha during luteolysis but towards PGE2 during the establishment of pregnancy in sheep. Biol. Reprod..

[7] Kim S., Choi Y., Spencer T.E., Bazer F.W. (2003). Effects of the estrous cycle, pregnancy and interferon tau on expression of cyclooxygenase two (COX-2) in ovine endometrium. Reprod. Biol. Endocrinol..

[8] Shirozu T., Iwano H., Ogiso T., Suzuki T., Balboula A.Z., Bai H., Kawahara M., Kimura K., Takahashi H., Rulan B. (2017). Estrous cycle stage-dependent manner of type I interferon-stimulated genes induction in the bovine endometrium. J. Reprod. Dev..

[9] Wei S., Zhang N., Zhang H., Chen Z., Li S., Wu W., Liu Z., Xia Z., Luo P., Cheng Q. (2026). Endoplasmic reticulum stress in disease pathogenesis: Its implications for therapy. Signal Transduct. Target. Ther..

[10] Lin P., Jin Y., Lan X., Yang Y., Chen F., Wang N., Li X., Sun Y., Wang A. (2014). GRP78 expression and regulation in the mouse uterus during embryo implantation. J. Mol. Histol..

[11] Lim W., Bae H., Bazer F.W., Song G. (2018). C-C motif chemokine ligand 23 abolishes ER stress- and LPS-induced reduction in proliferation of bovine endometrial epithelial cells. J. Cell. Physiol..

[12] Geng R., Ma C., Wen L., Dai W., Liu J., Yang J., Hu J. (2026). Echinacoside regulates the IRE1/XBP1 signaling pathway through HSP72 to reduce endoplasmic reticulum stress and improve diabetic kidney disease. Phytomedicine.

[13] Xu W., Yang Y., Li Y., Yang D., Wan S., Li N., Hua J. (2022). Spliced X-box binding protein 1 (XBP1s) protects spermatogonial stem cells (SSCs) from lipopolysaccharide (LPS)-induced damage by regulating the testicular microenvironment. Theriogenology.

[14] Lin T., Lee J.E., Oqani R.K., Kim S.Y., Cho E.S., Jeong Y.D., Baek J.J., Jin D.I. (2016). Tauroursodeoxycholic acid improves pre-implantation development of porcine SCNT embryo by endoplasmic reticulum stress inhibition. Reprod. Biol..

[15] Yoon S.-B., Choi S.-A., Sim B.-W., Kim J.-S., Mun S.-E., Jeong P.-S., Yang H.-J., Lee Y., Park Y.-H., Song B.-S. (2014). Developmental competence of bovine early embryos depends on the coupled response between oxidative and endoplasmic reticulum stress. Biol. Reprod..

[16] Guo S.-W., Yu W., Wang X., Liu J.-H., Zhang X.-Y., He C.-Q., Ding N.-Z. (2021). Expression and regulation of XBP1 in mouse uterus during early pregnancy. Sheng Li Xue Bao.

[17] Uzun A., Elçi Atılgan A. (2020). Is there a relationship between early pregnancy loss and maternal serum human X-box binding protein 1 level?. Med. Hypotheses.

[18] He W., Zhao Y., Yin L., Du Q., Ren W., Mao L., Liu A., Wang D., Qian J. (2025). The transcription factor XBP1 regulates mitochondrial remodel and autophagy in spontaneous abortion. Int. Immunopharmacol..

[19] Martell E., Kuzmychova H., Chawla U., Grewal A., Jain C., Venugopal C., Anderson C.M., Singh S.K., Sharif T. (2026). Glioblastoma cells that evade chemoradiotherapy-induced cell death exhibit a bifurcated glycolytic program. Cell Death Dis..

[20] Yuan Y., Xiao Y., Zou J., Luo L., Li M., Shen K., Wei L., Zhang Y., Wang P., Chen Y. (2026). Lactate derived from macrophages drives skin dermal fibroblasts phenotypic remodeling via MCT1-primed histone H3 lysine 23 lactylation in hypertrophic scar. Nat. Commun..

[21] Brown T.P., Ganapathy V. (2020). Lactate/GPR81 signaling and proton motive force in cancer: Role in angiogenesis, immune escape, nutrition, and Warburg phenomenon. Pharmacol. Ther..

[22] Jeong H., Kim R.-I., Koo H., Choi Y.H., Kim M., Roh H., Park S.G., Sung J.-H., Kim K.L., Suh W. (2024). Stem cell factor and cKIT modulate endothelial glycolysis in hypoxia. Cardiovasc. Res..

[23] Elia I., Rowe J.H., Johnson S., Joshi S., Notarangelo G., Kurmi K., Weiss S., Freeman G.J., Sharpe A.H., Haigis M.C. (2022). Tumor cells dictate anti-tumor immune responses by altering pyruvate utilization and succinate signaling in CD8^+^ T cells. Cell Metab..

[24] Zhang T., Chen L., Kueth G., Shao E., Wang X., Ha T., Williams D.L., Li C., Fan M., Yang K. (2024). Lactate’s impact on immune cells in sepsis: Unraveling the complex interplay. Front. Immunol..

[25] Feng Q., Liu Z., Yu X., Huang T., Chen J., Wang J., Wilhelm J., Li S., Song J., Li W. (2022). Lactate increases stemness of CD8 + T cells to augment anti-tumor immunity. Nat. Commun..

[26] Gao K., Yi Y., Xue Z., Wang Z., Huang S., Zhang B., Lin P., Wang A., Chen H., Jin Y. (2023). Downregulation of XBP1s aggravates lipopolysaccharide-induced inflammation by promoting NF-κB and NLRP3 pathways’ activation in goat endometrial epithelial cells. Theriogenology.

[27] Yang D., Jiang T., Liu J., Zhang B., Lin P., Chen H., Zhou D., Tang K., Wang A., Jin Y. (2018). CREB3 regulatory factor -mTOR-autophagy regulates goat endometrial function during early pregnancy. Biol. Reprod..

[28] Gao K., Zhao Y., Si M., Zhang B., Wang Z., Chen H., Lin P., Wang A., Jin Y. (2025). ERS regulates endometrial epithelial cell autophagy through XBP1s-mediated activation of the PI3K/AKT pathway. Sci. Rep..

[29] Zhang J., Zhao L., Li Y., Dong H., Zhang H., Zhang Y., Ma T., Yang L., Gao D., Wang X. (2022). Circadian clock regulates granulosa cell autophagy through NR1D1-mediated inhibition of ATG5. Am. J. Physiol. Cell Physiol..

[30] Zhang L., Liu X., Liu J., Ma L., Zhou Z., Song Y., Cao B. (2017). The developmental transcriptome landscape of receptive endometrium during embryo implantation in dairy goats. Gene.

[31] Gao K., Si M., Qin X., Zhang B., Wang Z., Lin P., Chen H., Wang A., Jin Y. (2025). Transcription factor XBP1s promotes endometritis-induced epithelial-mesenchymal transition by targeting MAP3K2, a key gene in the MAPK/ERK pathway. Cell Commun. Signal..

[32] Wang M., Gao Z., Zhao R., Zhou P., Chen J., Zhang H., Wang Y., Zhu W., Gao P. (2025). METTL14-Mediated M^6^A Modification of LINC01094 Induces Glucose Metabolic Reprogramming in Breast Cancer by Recruiting the PKM2/JMJD5 Complex. Adv. Sci..

[33] Li X., Hu L., Hu Q., Jin H. (2025). Lactate and lactylation in the kidneys: Current advances and prospects (Review). Int. J. Mol. Med..

[34] Olson G.S., Murray T.A., Jahn A.N., Mai D., Diercks A.H., Gold E.S., Aderem A. (2021). Type I interferon decreases macrophage energy metabolism during mycobacterial infection. Cell Rep..

[35] Wald M.E., Sieg M., Schilling E., Binder M., Vahlenkamp T.W., Claus C. (2022). The Interferon Response Dampens the Usutu Virus Infection-Associated Increase in Glycolysis. Front. Cell. Infect. Microbiol..

[36] Schafir A.L., Fernandez A.M., Fernández L.D.C., Materazzi L., Castagnola L., Hauk V., Irigoyen M., Cattaneo A., Gnocchi D., Tessari L. (2026). How decidualization dysregulation reshapes the nanomechanics of endometrial stromal cells. Mol. Hum. Reprod..

[37] English B.C., Savage H.P., Mahan S.P., Diaz-Ochoa V.E., Young B.M., Abuaita B.H., Sule G., Knight J.S., O’riordan M.X., Bäumler A.J. (2023). The IRE1α-XBP1 Signaling Axis Promotes Glycolytic Reprogramming in Response to Inflammatory Stimuli. mBio.

[38] Abdullah A., Talwar P., d’Hellencourt C.L., Ravanan P. (2019). IRE1α is critical for Kaempferol-induced neuroblastoma differentiation. FEBS J..

[39] Li X., Yang Y., Zhang B., Lin X., Fu X., An Y., Zou Y., Wang J.-X., Wang Z., Yu T. (2022). Lactate metabolism in human health and disease. Signal Transduct. Target. Ther..

[40] Wang X.-H., Xu S., Zhou X.-Y., Zhao R., Lin Y., Cao J., Zang W.-D., Tao H., Xu W., Li M.-Q. (2021). Low chorionic villous succinate accumulation associates with recurrent spontaneous abortion risk. Nat. Commun..

[41] Miyazawa H., Snaebjornsson M.T., Prior N., Kafkia E., Hammarén H.M., Tsuchida-Straeten N., Patil K.R., Beck M., Aulehla A. (2022). Glycolytic flux-signaling controls mouse embryo mesoderm development. eLife.

