# Supplementary material for: Transcription Factor XBP1s Impairs Endometrial Receptivity by Promoting ENO1-Mediated Glycolysis and Lactate Production
Source: Cells. 2026 Sep 14;15(18):1656. doi: 10.3390/cells15181656 (PMC13605876; doi:10.3390/cells15181656)

Figure S1. *ENO1* was successfully overexpressed and knocked down in gEECs. (A-C) Validation of ENO1 overexpression efficiency by Western blotting and RT-qPCR. **(D-F)** Validation of ENO1 knockdown efficiency by Western blotting and RT-qPCR. Data values are means ± SEM of three independent experiments. **P*<0.05; ***P*<0.01; ****P*<0.001.

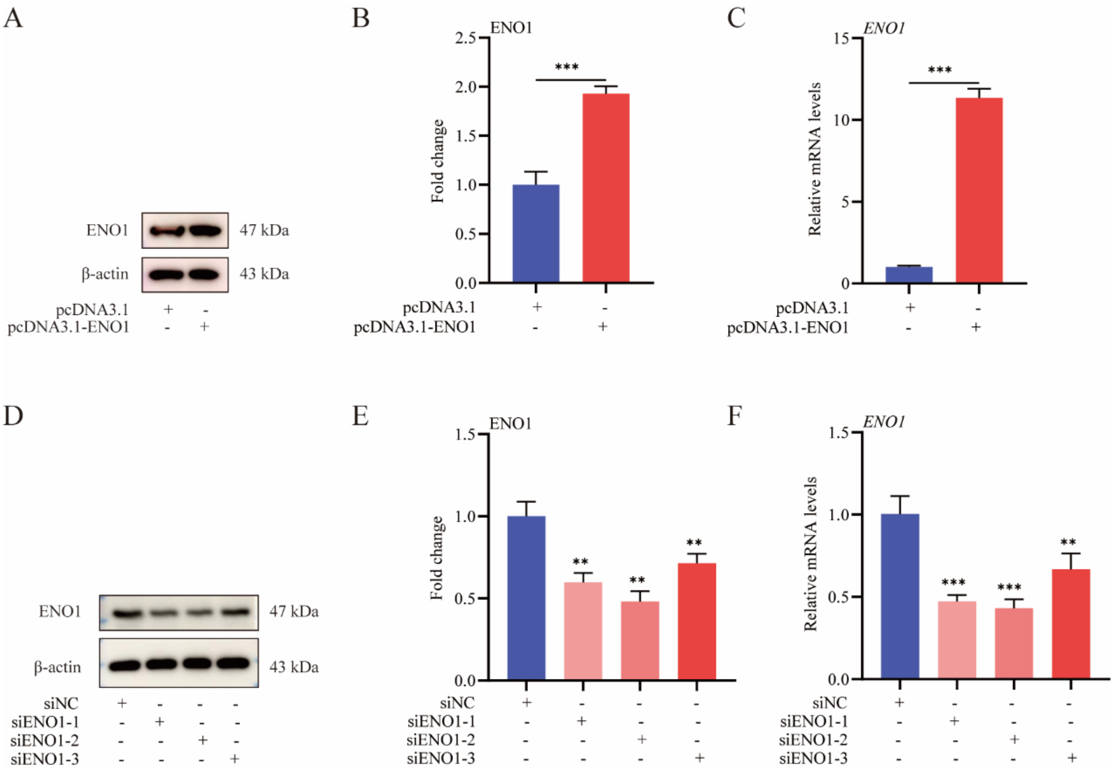

Supplement: Supplementary file 1 [file cells-15-01656-s001.zip › cells-4504682-supplementary.pdf]
